# The “GU-GU-RU” project to eliminate discrimination related to the health effects of the Fukushima nuclear accident

**DOI:** 10.1186/s12889-023-16883-2

**Published:** 2023-10-19

**Authors:** Isamu Amir, Yuichiro Eguchi, Kousaku Saotome, Soichiro Ogawa, Yoshiyuki Kojima, Tomoaki Tamaki, Masaharu Tsubokura

**Affiliations:** 1 Department of Radiation Health Management, Fukushima Medical University School of Medicine, 1 Hikariga-oka, Fukushima-city, 960-1295 Fukushima Japan; 2 Loco-Medical General Institute, 1178-1 Kanada, Mikatsuki-cho, Ogi-city, 845-0032 Saga Japan; 3 Department of Radiological Sciences, Fukushima Medical University School of Health Sciences, 10-6 Sakae-machi, 960-8516 Fukushima-city, Fukushima Japan; 4 Department of Urology, Fukushima Medical University School of Medicine, 1 Hikariga-oka, Fukushima-city, 960-1295 Fukushima Japan; 5 Department of Health Risk Communication, Fukushima Medical University School of Medicine, 1 Hikariga-oka, Fukushima-city, 960-1295 Fukushima Japan

**Keywords:** Fukushima nuclear accident, Radiation exposure, Health issues, Genetic effects, Discrimination, Prejudice, Government project

## Abstract

**Background:**

Although 12 years have passed since Great East Japan Earthquake and following Fukushima nuclear accident, approximately 40% of Japanese citizen still believe that the current radiation exposure in Fukushima residents will likely/ very likely to cause genetic effects of radiation. This incorrect understanding could continue unexpected discrimination and prejudice towards those from Fukushima now and in the future. In order to provide updated knowledge and eliminate rumors related to radiation, Japanese Ministry of the Environment has launched “GU-GU-RU” project in 2021 with consisting of five sections.

**Objective:**

(1) To discuss the objectives and effects of the “GU-GU-RU” project (results after the first year), (2) to present administrative measures that may be effective in the long-term to prevent unjustified discrimination and prejudice, and (3) to eliminate rumors in the event of future large-scale disasters, including radiation disasters.

**Methods:**

We showed the contents of each sections carried out under the project and observed the result of first-year activities in each section.

**Results:**

Among the programs, the “Radiation College” has steadily produced positive results, with nearly 1,300 students participating and 50 students sharing their thoughts and ideas. In addition, the project has adopted strategies such as creating and broadcasting a TV program and collaborations with manga, which are expected to have a significant impact on society.

**Conclusions:**

Compared to previous efforts on disseminating information related to health effect of radiation exposure, the “GU-GU-RU” project has taken a different approach in providing primary data of radiation and its health effects, which could become a better understanding of health effects of radiation for the general public, in order to eliminate rumors that may lead unjustified discrimination and prejudice.

## Background

Racial and gender prejudice and social discrimination have always been present in society. Social movements, such as the civil rights movement in the US from the 1950 to 1970 s, have attempted to address these inequalities.

From a public health perspective, after an epidemic of a novel infectious disease (such as HIV/AIDS or coronavirus disease 2019 [COVID-19]), discrimination has been concentrated against affected individuals and their families owing to the fear of infection. Indeed, it takes time for people to come to a correct understanding of infectious diseases and learn not to discriminate against affected individuals.

The health effects following radiation disasters are similar to those of infectious diseases. Long-term and secondary psychological effects (such as discrimination, prejudice, and stress) related to radiation can occur owing to changes in people’s social life and environment, in addition to the direct effects of radiation exposure on the body [1, 2]. In Japan, discrimination took place against residents who had evacuated from Fukushima, and bullying at schools and other places occurred following the accident at the Fukushima Daiichi Nuclear Power Station (FDNPS) of the Tokyo Electric Power Company after the Great East Japan Earthquake (GEJE) happened in 2011 [3]. We consider that psychological effects of the events described above are considerably greater than the mere physical effects of radiation exposure; therefore, we believe that a comprehensive understanding of these incidents and countermeasures to address them is a significant public health issue. However, sufficient measures have not yet been established to direct the long-term response.

The United Nations Scientific Committee on the Effects of Atomic Radiation (UNSCEAR) has reported on the health effects of radiation exposure from the FDNPS accident, stating, “No adverse health effects among Fukushima residents have been documented that are directly attributable to radiation exposure from the FDNPS accident. The Committee’s revised estimates of dose are such that future radiation-associated health effects are unlikely to be discernible” [4]. On the one hand, since this report does not state that there are no radiological effects on local residents who exposed radiation after FDNPS accident, we should keep focusing on the fact if stochastic effect could happen in the future. On the other, according to Fukushima Health Management Survey (FHMS) conducted by Fukushima Medical University, it reports that genetic effect of neonates or infants in Fukushima prefecture has not increased after the accident until 2019. However nearly 40% of the Japanese public believe that the health effects on the next generation of Fukushima residents due to current radiation exposure are likely/ very likely to occur, which could lead to discrimination or prejudice towards those from Fukushima [5–7].

Based on the current situation, Japanese Ministry of the Environment (MOE) launched the “GU-GU-RU” project in July 2021 to dispel incorrect understanding, discrimination, and prejudice associated with the health effects of radiation [8]. In order to achieve the designated goal, which is to reduce the proportion of those who have incorrect perception related to health effects of radiation among Japanese citizen from 40 to 20%, this project determined five sections, which are “to know,” “to learn,” “to make decisions,” “to listen,” and “to research.” However, to date, no academic report has presented the details and effects of this project. A discussion of the effects of this project, presenting the observations made related to each objective, would be useful and provide important guidance when considering future measures to deal with long-term health effects and other issues after a large-scale disaster.

The purpose of this paper is to discuss the objectives and effects of the “GU-GU-RU” project (results after the first year), to present administrative measures that may be effective in the long-term to prevent unjustified discrimination and prejudice, and to eliminate rumors in the event of future large-scale disasters, including radiation disasters.

## Methods (activities)

The “GU-GU-RU” project is conducted by MOE with the aim to create an occasion where people can learn to “understand and interpret information” and “judge and decide without being misled by rumors.” Specifically, this project aims to update knowledge related to the health effects of radiation and dispel incorrect understanding that may lead to discrimination and prejudice. The project title, “GU-GU-RU,” was derived from the last letters of the Japanese verbs describing the three main pillars of the project, which are “learning facts and building knowledge (Tsumu-GU),” “connecting people, community, and society (Tsuna-GU),” and “messages are transmitted as an individual matter (Tsutawa-RU).”

MOE aims to reduce the proportion of Japanese citizens who believe that the current radiation exposure in Fukushima residents is likely/ very likely to cause genetic effects, from 40% in fiscal year 2020 to 20% by the end of fiscal year 2025, which is the end of March 2026.

The kick-off meeting was held on July 15, 2021. The Minister of the Environment, MP Shinjiro Koizumi, attended the event, and it was covered by media outlets [9, 10]. Fukushima Medical University is actively participating in the project, holding various seminars and recording sessions to fulfill its role as a medical institution that promotes the healthcare of local Fukushima residents [11].

**Fig. 1:**
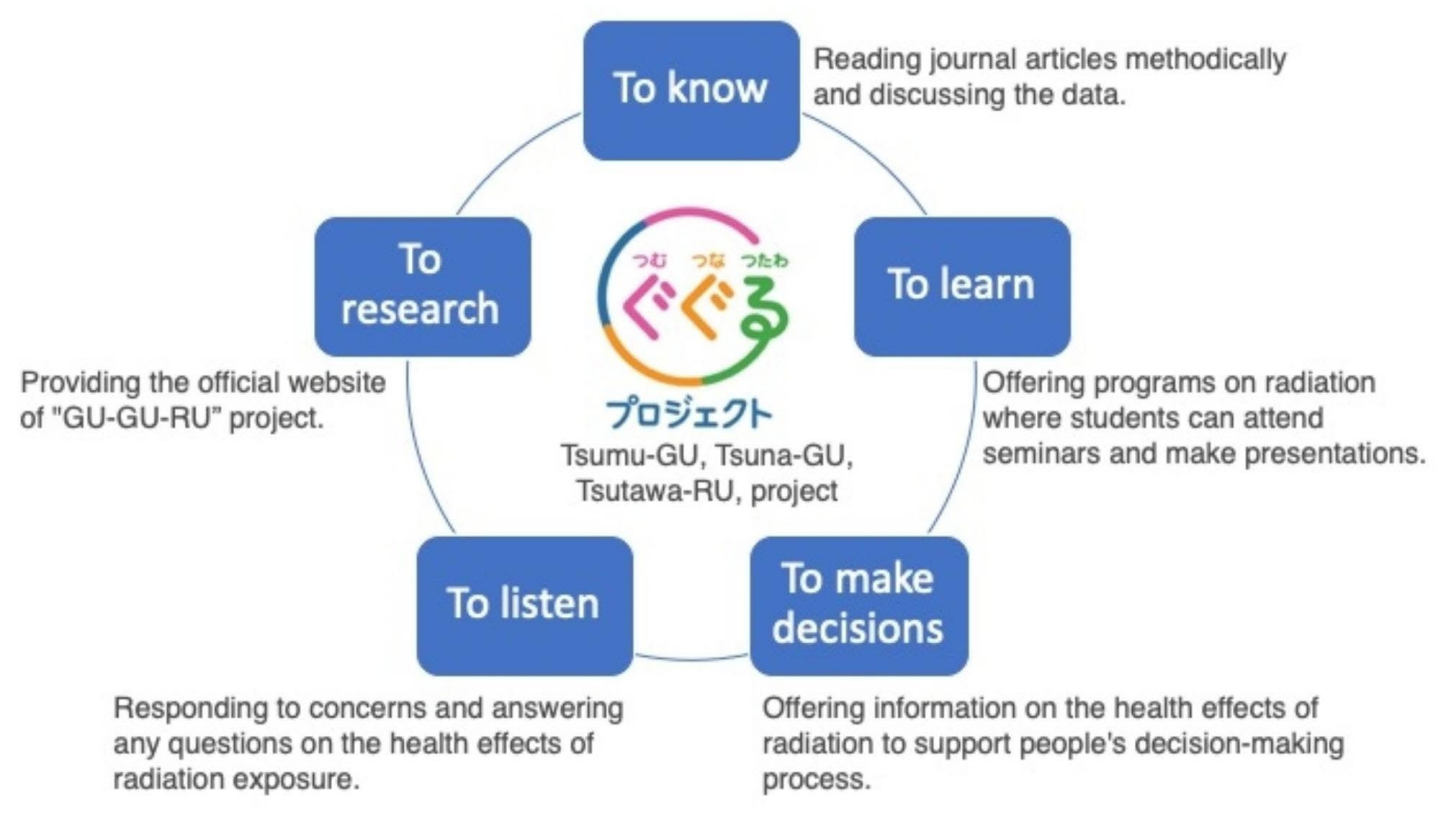
Five sections of the “GU-GU-RU” project

The “GU-GU-RU” project consists of five sections, which are “to know,” “to learn,” “to make decisions,” “to listen,” and “to research” (Fig. 1). These were set at the kick-off meeting in 2021 and are outlined below.

(1) “To know” (reading academic papers scientifically).

This program provides students an opportunity to learn “how to read and write academic papers,” using published journal articles as reference material. This program also includes a review of how we perceive information from media and social networking services.

For example, an article on the Fukushima nuclear accident and the corresponding increase in the number of pediatric patients with congenital heart diseases has been published in an academic journal. The workshop explains the study’s data acquisition methods and logic development using the data presented in the article and describes how the paper can be validated using its data sources. The session teaches participants how to interpret and understand this information [12–15].

(2) “To learn” (Radiation College).

This program includes seminars and other events for universities nationwide, providing an opportunity to learn the basic scientific knowledge about radiation and its health effects, as well as an opportunity to present what they have learned. Students can take part in face-to-face seminars at universities or via online videos. MOE also holds an event for those who wish to present what they have learned through this program. This is divided into the “presentation section” and the “dialog writing section.”

In the presentation section, applicants can present their research and compilation regarding the health effects of radiation by expressing in their own words. They can choose to be recorded either by professionals or by themselves. The presentations are evaluated later by an outside panel of judges, who select the winners of the Excellence Award.

In the dialog writing section, applicants can create a dialog on discrimination, prejudice, and scientific facts along with a designated scenario (for example, a woman presenting knowledge to her parents, who are concerned about the genetic effects of radiation in the context of her marriage to a male radiologist). An Excellence Award is selected in the same way as in the presentation section, and the dialog receiving the award is actually dramatized.

(3) “To make decisions”.

This project provides information on topics such as the health effects of radiation so that individuals can make their own decisions with confidence based on the concept of “information provision and decision making.”

After the Chernobyl nuclear power station accident occurred in 1986, the incidence of thyroid cancer increased among children after 4 years of the accident due to internal exposure to radioactive iodine, which was one of the health effects associated with radiation [16]. In the FDNPS accident, the radiation dose received by infants and children (which was mainly radioactive iodine released from the containment vessel of the nuclear reactor) was low, and the resulting health effects were not as serious or severe as those after the Chernobyl accident [4]. However, the people of Fukushima Prefecture had a growing concern that a situation like the Chernobyl accident might occur again.

Meanwhile, Fukushima Prefecture began the “Fukushima Health Management Survey (FHMS)” in 2011 to monitor the health of Fukushima residents [17]. The FHMS provides thyroid screening examinations for approximately 380,000 people who ranged in age from fetuses to the age of 18 at the time of the accident [18].

The purpose of this program is to provide appropriate information and create an environment where individuals eligible for thyroid screening can make their own decisions whether to undergo an examination. This concept was derived from questions on whether the decision-making process for those eligible for thyroid screening was being carried out appropriately.

(4) “To listen”.

The purpose of this program is to expand and improve the system that enables residents of Fukushima who are anxious about radiation to receive a consultation about radiation-related issues.

One of the goals for this program is to strengthen the activities of the “Radiation Risk Communication Consultant Support Center” established by MOE [19]. Another function of the program is to provide risk communication activities and consultation on radiation to residents of Fukushima who are anxious about radiation under the concept of “to pay attention closely to concerns and questions.”

(5) “To search”.

This program aims to reduce anxiety about radiation health effects by presenting information on the official website of the project. The website can be used like a dictionary to search for data whenever people are concerned about something related to radiation. The website is constructed based on the contents of a booklet titled, “Basic Information Regarding Health Effects of Radiation”, and the “Portal Site on Radiation Health Effects” [20, 21].

## Results

After the kick-off meeting on July 15, 2021, each project launched and obtained the following results:

(1) and (2) “To know” and “To learn”.

**Fig. 2:**
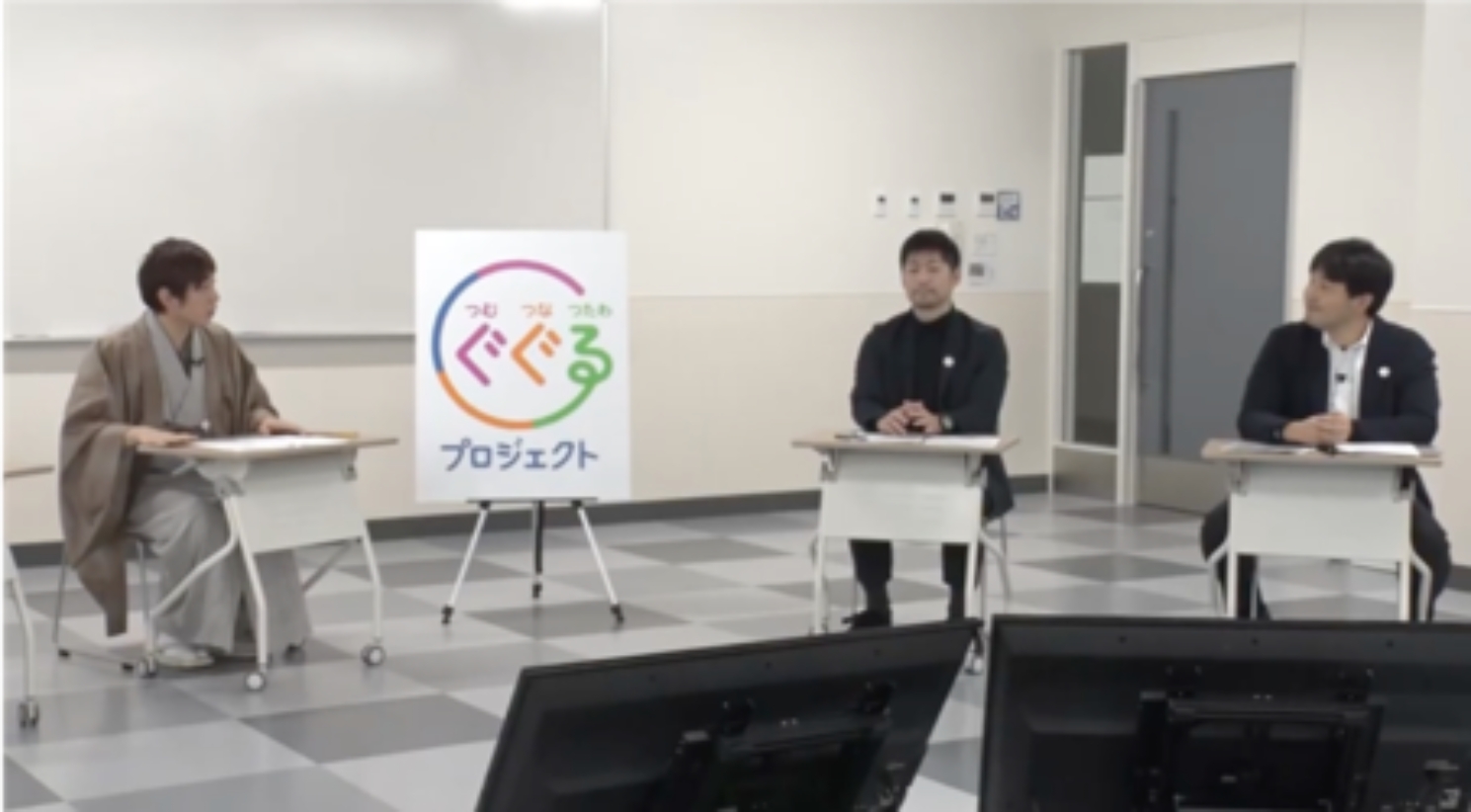
Public seminar held at Fukushima Medical University (special dialog)

In the first year of the project, the program integrated “to know” and “to learn.” Specifically, “Radiation College” seminars were held at 49 universities and 1 high school throughout Japan, with 1,345 students participating. The seminar contents were primarily (i) basic knowledge of radiation and (ii) the credibility of data published in articles, which were related to the content of “to know.” In December 2021, a public seminar (special dialog) was held at Fukushima Medical University in conjunction with the Radiation College seminar (Fig. 2).

The presentations and dialogs submitted by students were reviewed by outside experts, and an award ceremony was held on February 28, 2022, at the “GU-GU-RU” project forum. Six students received awards for their outstanding work. Among the winners in the presentation section, one student stated, “I cannot shake off the fear that prejudice will be passed on to my children and ruin their lives,” and another warned that “decreased interest will cause individuals to fixate on past information and have limited access to the latest information.” Another cited the UNSCEAR 2020/2021 report, pointing out that incorrect understanding about the health effects of radiation and rumors associated with the lack of updated and correct knowledge still persist.

In the dialog writing section, one piece addressed excessive anxiety about radiation owing to ambiguous information and feelings of being subjected to discriminatory re-marks in a profession that deals with radiation. Another presented the truth of what is written in academic papers, and the other addressed the lack of genetic effects on the second generation exposed to the atomic bomb.

In addition, on March 4, 2022, a “Nikkei Seminar” was held online for business people in a tie-up with the Nihon Keizai Shimbun (Japan Economic Times). The seminar lectures included a viewpoint from behavioral economics. More than 500 people were estimated to have watched the seminar. The following six videos from the Radiation College are currently posted on the official website of the project explained in the activity Sect. (5).

(1) Presentation category entries (all presentations) [22].

(2) Short animation in the dialog creation section (excellent works) [23–25].

(3) Special dialog (two Fukushima Medical University faculty members and rakugo storyteller Sanshiro Katsura) [26] (Fig. 2).

(4) Video library [27–29].

(5) Reconstruction Agency Video [30].

(6) Nikkei Seminar Video [31].

(3) “To make decisions”.

**Fig. 3:**
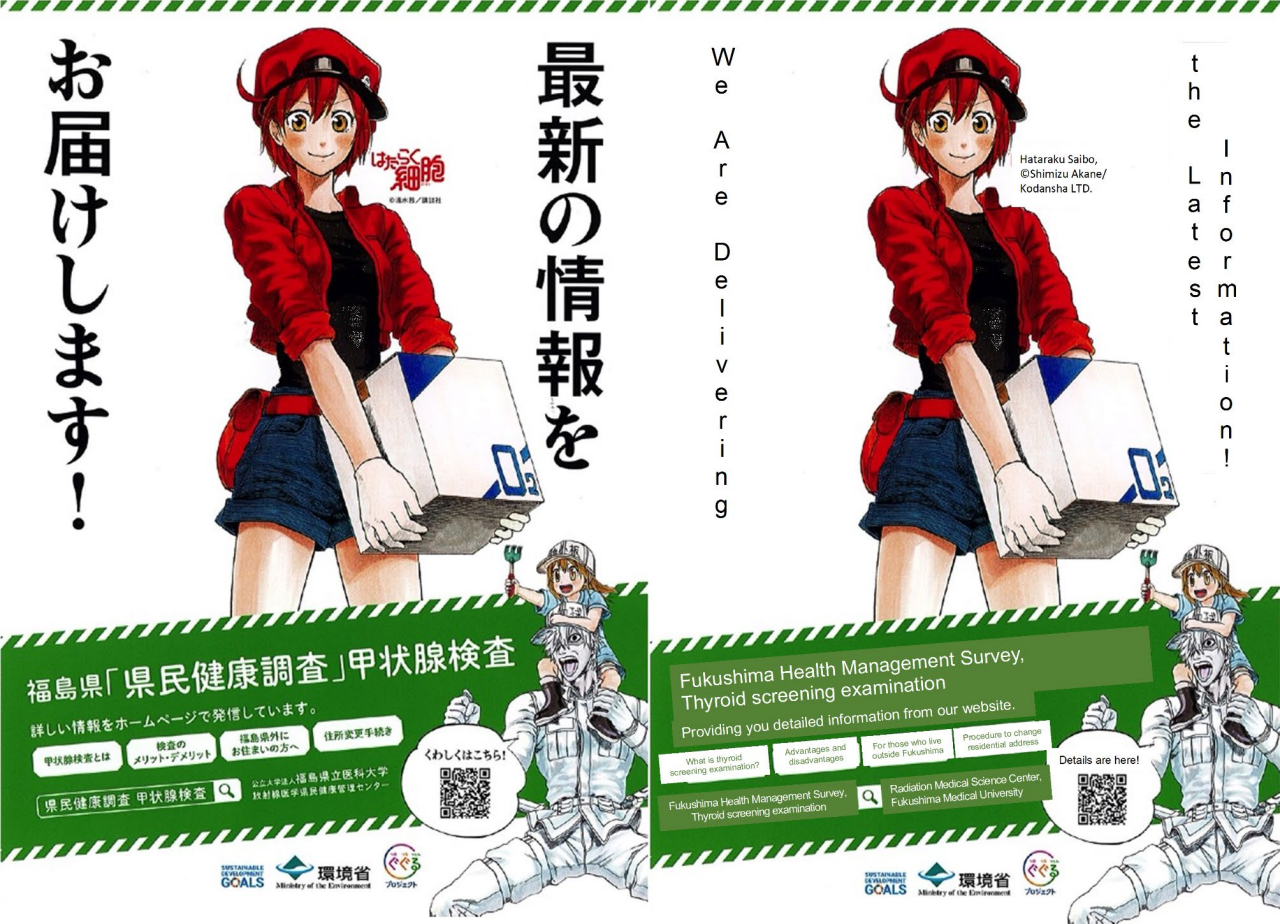
A poster of “Hataraku Saibo” (Cells at Work!), noticing the website providing the latest information related to the thyroid screening examination (left: original Japanese version, right: English version translated by the first author)

The purpose of this program is to provide decision-making information for the people eligible for thyroid screening examination (aged 11 or older as of 2021) through the FHMS. As a first step, a poster and a clear file folder using a manga “Hataraku Saibo” (Cells at Work!, ©Shimizu Akane/ Kodansha LTD) were created and delivered in Fukushima Prefecture [32] (Fig. 3). “Hataraku Saibo” is a manga that illustrates various physiological phenomena that occur in the human body from the viewpoint of cells, in which anthropomorphic red blood cells, along with white blood cells and platelets, are the main characters. The poster and clear file folder indicate the websites showing the latest information on thyroid screening examination and recruiting medical organizations for examination, which can be easily accessed using a QR code.

In addition, a leaflet describing the thyroid screening examination provided by FHMS was prepared and distributed to those eligible for thyroid screening examinations [33]. It provides basic information about undergoing a thyroid examination, including the radiation dose of radioactive iodine for Fukushima Prefecture residents due to the FDNPS accident, and the relationship between thyroid cancer detected in previous examinations and radiation exposure. Moreover, it explains the rights that individuals eligible for thyroid examinations have to make decisions before undergoing the examinations (for example, deciding whether to take the examination, ask questions regarding the examination, and postpone to undergo the examination). These explanations and messages are described with illustrations.

**Fig. 4:**
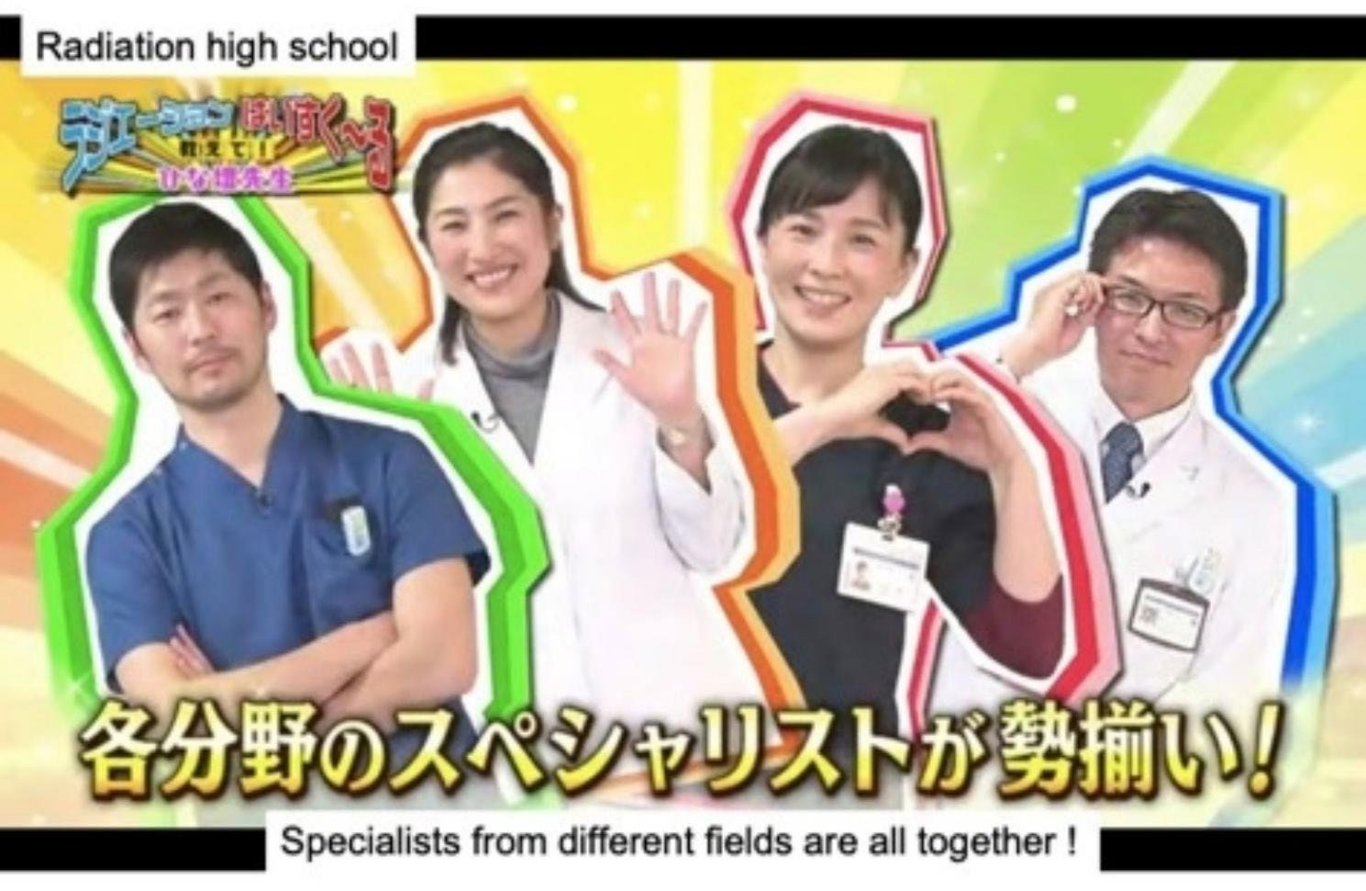
A scene from a TV program dedicated to the use of radiation in medical practice (English subtitles were added by the first author)

Additionally, information on the use and application of radiation in the medical field and radiation protection was disseminated through the production and broadcast of a TV program focusing on radiation-related professions (medical radiologists and radiology technicians enrolled at Fukushima Medical University and Kyoto Medical University) [34] (Fig. 4).

(4) “To listen”.

In this program, students participating in the recording session for the presentation section of “Radiation College” had an opportunity to hear from returned residents after the evacuation order was lifted. Topics students heard were the local government’s response at the time of the GEJE and following FDNPS accident, as well as reconstruction efforts to date [35]. The students exchanged opinions on measures at the municipal level to promote the return of residents who had evacuated and gained new insights. They also visited the Great East Japan Earthquake and Nuclear Disaster Memorial Museum to learn about the actual damage caused by the earthquake and tsunami, and the evacuation following the nuclear accident [36].

**Fig. 5:**
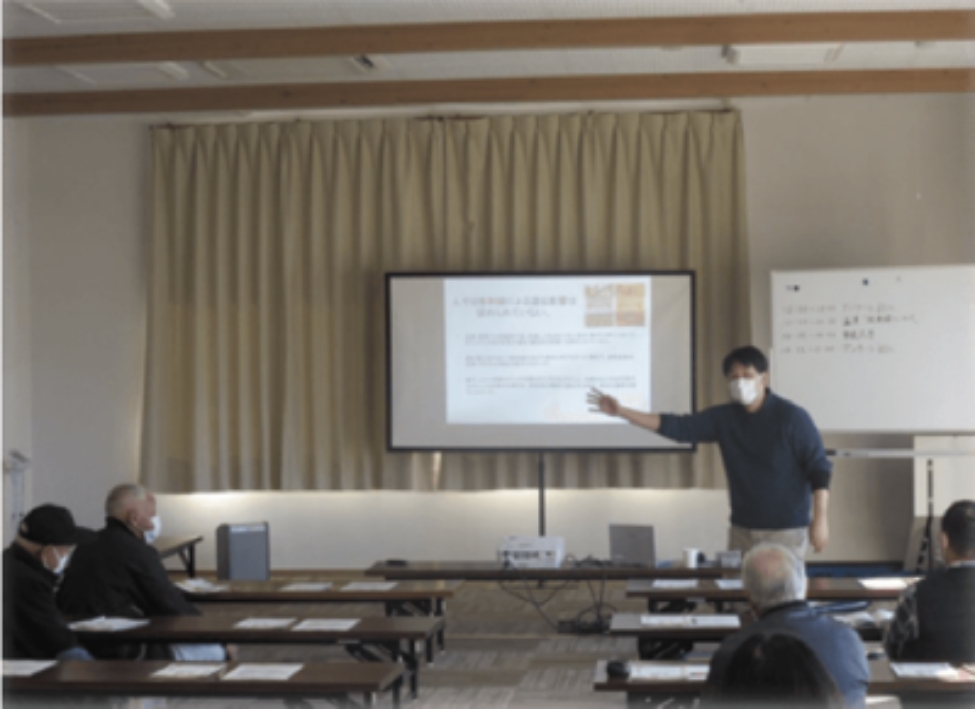
A lecturer from Fukushima Medical University is providing information relevant to radiation

The other study session, which a faculty member from Fukushima Medical University led out in, was held for local residents to provide information on current radiation levels in areas where the evacuation order is expected to be lifted in the future and to listen to their concerns [37] (Fig. 5). Participants raised questions about the difference between exposure to the atomic bombs unleashed in Hiroshima and Nagasaki and exposure to the FDNPS accident. Indeed, they asked the accumulation of radiation in the body from daily exposure, and the radiation levels contained in the natural mushrooms they eat daily. A faculty member from Fukushima Medical University answered these inquiries.

(5) “To research”.

In this project, various information was posted on the official “GU-GU-RU” project website. Examples of the information include the results of a questionnaire survey on the health effects of radiation, a link to the statement from MOE regarding the letter issued on January 27, 2022, by five former Japanese Prime Ministers to the President of the European Union, which was to clarify the misunderstanding of the causal relationship between radiation exposure and thyroid cancer [38, 39]. This page has been continuously updated so that individuals who are concerned about the health effects of radiation can easily access it as a reliable source of information. In addition, a link to the English version of the “Portal Site on Radiation Health Effects, etc.” has been established to provide overseas access [40].

## Discussion

Now that we have introduced and summarized the five sections of the “GU-GU-RU” project, we will discuss the characteristics of the project from four different perspectives.

(1) Purpose.

In the past, risk communication and many projects related to the health effects of radiation have aimed to provide residents with scientifically correct data about radiation. The focus has been concentrated on scientific content, such as types of radiation and the health risks associated with radiation exposure. The communication method has often been in the form of lectures, which is a one-way provision of scientific facts from the lecturers (experts) to the target audience (residents).

Meanwhile, in the “GU-GU-RU” project, the objective of “dispelling incorrect understanding, discrimination, and prejudice” has been given priority over the scientific content. This includes people’s sense of morality, social norms, and values. Scientific content can never be 100% accurate, as new findings may always emerge, and their interpretation can change. However, morality, social norms and values are easily understood among the general public because they are historically, educationally, and culturally embedded in the Japanese society.

It is believed that the project participants themselves realize and make it personal that incorrect understanding about radiation are unconsciously related to discrimination and prejudice. Indeed, we can recognize that participants can achieve the goals of the project by realizing that updating knowledge.

(2) Targeting.

In large-scale, government-led projects such as the “GU-GU-RU” project, the target audience must always be considered, and it is difficult to measure the effectiveness of the project. Although scientific knowledge on radiation has been provided in past projects, it is questionable whether the Japanese public has understood it. This is evidenced by the fact that 40% of the Japanese citizens still believe that the genetic effects of radiation exposure on the next generation is likely, very likely to occur [38].

This result made it necessary to consider a different approach. Based on the concepts of behavioral economics and social marketing, the “GU-GU-RU” project has set its target as the generation (teens to 30s) who will be facing life events in the near future, such as entering university or vocational school, finding and beginning a job, getting married, becoming pregnant, giving birth, and raising children. This target was set for three following reasons.

First, risk communication and projects related to radiation have been less effective in the past because they targeted people of all ages, thus blurring the target audience when creating content and forming strategies.

Second, teens to 30s are the generation most likely to suffer from discrimination and prejudice since 40% of the general public believe the “genetic effects associated with radiation exposure” is likely/ very likely to occur, as indicated at the beginning of this article. Their acquisition and appropriate dissemination of correct knowledge, even if they are subjected to discrimination and prejudice, must be accompanied by the ability to present data with evidence and explain it from their own perspective.

Third, the program encourages decision making by providing accurate information on radiation-related health effects. Teenagers to those in their thirties face a variety of life events described before, and each of these events is accompanied by decision making. For those who are anxious about radiation exposure or its subsequent effects, this project attempts to alleviate their anxiety and promote appropriate independent decision making by providing accurate information and options.

Despite these assumptions, the circumstances surrounding the “GU-GU-RU” project are becoming different. Therefore, the project is progressing through a process of trial and error in a constantly changing situation.

(3) Encouraging proactive action.

One feature of the project is promoting proactive or active action for participants, rather than passive acceptance of information.

For example, in the section of “to know,” the seminars are not only showing and explaining articles and their contents but also introducing contrast in articles that raise doubts about the genetic effects associated with radiation exposure after the FDNPS accident, along with verification papers and explanations of what parts of the data and logic of the papers are controversial.

Finally, the seminars ask participants to verify the data presented to them from their own perspectives, rather than taking it for granted under “reliability of information.” This could apply not only for the data related to health effects of radiation exposure, which is the main part of “GU-GU-RU” project, but also for all the information that surround us, which could be applicable for our daily lives.

In fiscal year 2021, 50 students participated in a presentation section and a dialog writing section as opportunities to communicate their thoughts and ideas (Students who participated in both divisions are counted as one).

In addition, it is also important to note that presentations and dialogs created by students are evaluated by experts, which motivate them to re-think about their own expression styles or techniques, which may lead to better works. Indeed, videos of the students’ own presentations and dialogs are posted on the “GU-GU-RU” project website, which may encourage others who desire to participate to further refine their high-quality thoughts and ideas. One of the main features of the “GU-GU-RU” project is that it provides an opportunity for students who wish to share their own views on the health effects of radiation (an occasion to express their ideas). We think that continuous opportunities for students who take part in such events will be necessary.

(4) General discussion.

Since more than 12 years have passed since the GEJE and FDNPS accident, memories of the disaster are fading among the Japanese people. However, we should not overlook the growing indifference that has resulted from the accident disappearing from the minds of individuals. This is because indifference on the part of individuals is a major factor preventing the “updating of information” indicating one of the goals of the “GU-GU-RU” project.

The background of this phenomenon is not simple. After the GEJE and subsequent FDNPS accident in 2011, natural disasters (earthquakes, typhoons, volcanic eruptions, etc.) have occurred every year in Japan. Indeed, the COVID-19 pandemic was one of the health issues people greatly paid attention to. These incidents constantly overwrite people’s memories of other natural disasters and health crises. As a result, the awareness of the GEJE and FDNPS accident has become relatively low among the public, as their memories diminish or they gradually become indifferent. Consequently, 40% of the Japanese public still has incorrect understanding about the health effects of radiation.

Particularly, the impact of the COVID-19 pandemic, which has been ongoing since 2020, has spread throughout Japan, transforming our way of life in terms of limited medical care, restrictions on daily activities, and continuous wearing of masks. The social impact of COVID-19 was greater than that of the GEJE, and information on COVID-19 took precedence over memories of the GEJE and FDNPS accident, accelerating the declining of such memories among the general public.

However, it is necessary to shed light on discrimination and prejudice toward those from Fukushima regarding the health effects of radiation through the “GU-GU-RU” project, which must not be abandoned. Therefore, we need to update people’s knowledge by providing and disseminating the latest details of scientific facts steadily, and the current situation of Fukushima, which could revive people’s fading consciousness of the situation. This is a necessary task to eliminate discrimination and prejudice, which are based on incorrect understanding of radiation exposure relevant to the FDNPS accident.

To think about the project in the future, on the one hand, it is important to try to eliminate discrimination and prejudice through updating knowledge of health effect of radiation for participants. On the other, we need to show them the possibility of stochastic effect of radiation with mathematical and scientific perspectives. Indeed, we have to explain the significance of radiation safety and radiation protection implemented at medical fields, which is necessary for us, and not to underestimate them.

## Conclusion

The “GU-GU-RU” project was initiated to provide updated knowledge related to radiation and to eliminate rumors that may lead to unjustified discrimination and prejudice regarding the health effects, especially genetic effects, of radiation. The goal of the project is to reduce the proportion of Japanese citizens nationwide who believe that the current radiation exposure in Fukushima residents will likely cause genetic effects of radiation, from 40 to 20% by the end of March 2026.

The kick-off meeting held in July 2021 was covered by the press, and the project attracted a great deal of public attention. Among the programs, the “Radiation College” has steadily produced positive results, with nearly 1,300 students participating and 50 students sharing their thoughts and ideas. In addition, the project has adopted strategies such as creating and broadcasting a TV program and collaborations with manga, which are expected to have a significant impact on society.

Compared to previous activities, the “GU-GU-RU” project has taken a different approach in providing information related to radiation and its health effects. The project incorporates the perspective of behavioral economics and takes a proactive approach to the media. Each program have been carried out with its own unique characteristics, such as collaboration with manga. Although the project has just begun, it is difficult to obtain the prompt result in terms of percentage decrease after the first year [41]. Therefore, a continued development for this project to achieve the goal by the end of March 2026 is expected.

## Data Availability

The datasets used and/or analyzed during the current study available from the corresponding author on reasonable request.

## Abbreviations

FDNPS: Fukushima Daiichi Nuclear Power Station
FHMS: Fukushima Health Management Survey
GEJE: Great East Japan Earthquake
MOE: Japanese Ministry of the Environment
UNSCEAR: The United Nations Scientific Committee on the Effects of Atomic Radiation
